# Cell Substratum Adhesion during Early Development of *Dictyostelium discoideum*

**DOI:** 10.1371/journal.pone.0106574

**Published:** 2014-09-23

**Authors:** Marco Tarantola, Albert Bae, Danny Fuller, Eberhard Bodenschatz, Wouter-Jan Rappel, William F. Loomis

**Affiliations:** 1 Max Planck Institute for Dynamics and Self-Organization (MPIDS), Laboratory for Fluid Dynamics, Pattern Formation and Biocomplexity, Goettingen, Germany; 2 Center for Theoretical Biological Physics, University of California San Diego, La Jolla, California, United States of America; 3 Institute of Nonlinear Dynamics, Georg-August University, Goettingen, Germany; 4 Laboratory of Atomic and Solid-state Physics, Cornell University, Ithaca, New York, United States of America; 5 Department of Physics, University of California San Diego, La Jolla, California, United States of America; 6 Cell and Developmental Biology, Division of Biological Sciences, University of California San Diego, La Jolla, California, United States of America; Université de Genève, Switzerland

## Abstract

Vegetative and developed amoebae of *Dictyostelium discoideum* gain traction and move rapidly on a wide range of substrata without forming focal adhesions. We used two independent assays to quantify cell-substrate adhesion in mutants and in wild-type cells as a function of development. Using a microfluidic device that generates a range of hydrodynamic shear stress, we found that substratum adhesion decreases at least 10 fold during the first 6 hr of development of wild type cells. This result was confirmed using a single-cell assay in which cells were attached to the cantilever of an atomic force probe and allowed to adhere to untreated glass surfaces before being retracted. Both of these assays showed that the decrease in substratum adhesion was dependent on the cAMP receptor CAR1 which triggers development. Vegetative cells missing talin as the result of a mutation in *talA* exhibited slightly reduced adhesive properties compared to vegetative wild-type cells. In sharp contrast to wild-type cells, however, these *talA* mutant cells did not show further reduction of adhesion during development such that after 5 hr of development they were significantly more adhesive than developed wild type cells. In addition, both assays showed that substrate adhesion was reduced in 0 hr cells when the actin cytoskeleton was disrupted by latrunculin. Consistent with previous observations, substrate adhesion was also reduced in 0 hr cells lacking the membrane proteins SadA or SibA as the result of mutations in *sadA* or *sibA*. However, there was no difference in the adhesion properties between wild type AX3 cells and these mutant cells after 6 hr of development, suggesting that neither SibA nor SadA play an essential role in substratum adhesion during aggregation. Our results provide a quantitative framework for further studies of cell substratum adhesion in *Dictyostelium*.

## Introduction

Motile cells must have traction on the substratum to extend the anterior pseudopod and retract the rear. While the cytoskeleton generates the protrusive and contractile forces, the interaction of the cell surface with the underlying support is necessary to transmit the forces. Many mammalian cells carry integrin proteins in their membranes that can bind to fibronectin, laminin, vitronectin or collagen that form extracellular matrices [1]–[4]. Inside the cell, the cytoplasmic face of heterodimeric integrins associates with various actin-binding proteins, such as talin, α-actinin, paxillin, vinculin or filamin. They tether to the F-actin of the cytoskeleton forming focal adhesions that remain fixed to the matrix until the cell has moved over them. While much is known about the structure and function of focal adhesion complexes of mammalian cells binding to proteins of the extracellular matrix, it is becoming clear that such specialized complexes are not always necessary for cell substrate adhesion and motility [4], [5].

The *Dictyostelium* genome does not carry genes for integrins or any of the extracellular matrix proteins [6]. Moreover, *Dictyostelium* cells are able to move with normal speeds on naked glass, even in the presence of liquid flow that would be expected to wash away secreted materials. Substratum adhesion does not appear to rely on covalent, ionic or hydrogen bonds since cells are also able to adhere and move equally well on hydrophobic silanized glass or hydrophilic serum albumin coated glass [7]–[10]. It has been proposed that Van der Waals attractive forces between cell surface glycoproteins and the underlying substratum are sufficient to account for the adhesion of these cells [9].

Nevertheless, cells lacking a specific surface protein, SibA, were shown to have reduced contact area, as well as adhesion and phagocytosis defects [11]. The stability of SibA as well as its expression on the surface was found to be dependent on two other membrane proteins, SadA and Phg1A [12]. Several studies also showed that cells lacking SadA, Pgh1A and talin had reduced substratum adhesion [10]–[19]. However, all of these studies were carried out with 0 hr cells. It is not clear whether these proteins also function in substratum adhesion during development. In this study, we quantify cell substratum adhesion using two independent assays. The first assay measures the fraction of adherent cells in a microfluidic device that is able to generate a range of hyndrodynamic shear stress. The second assay determines the adhesive force between a single cell and the substratum using an Atomic Force Microscope (AFM). We quantified the adhesive properties of wild-type and several mutant strains as a function of development and found that both assays gave consistent results.

## Results

### Adhesion during early development

**Figure 1:**
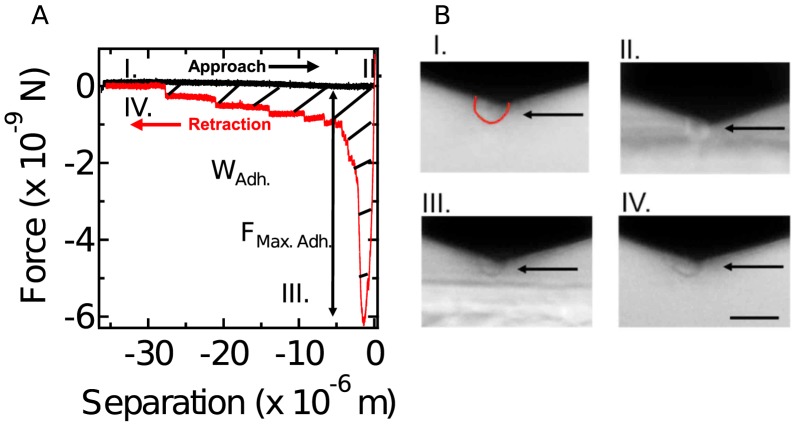
Single Cell adhesion Force Spectroscopy assay. **A.** Force was measured from bending of the cantilever and a typical force-separation curve is shown for an approach-retraction cycle highlighting the two assessed parameters: maximum adhesion force F_Max.Adh._ and the work of adhesion W_Adh._ (integral of the hatched area). I–IV refers to the four panels shown under B. **B.** Side view of a cycle of approach and retraction of a cell attached to a cantilever. Panels I and II: A cell can be seen hanging below the cantilever as it approaches the glass slide (light colored surface, *Dictyostelium* contour in red under I). Panels III and IV: The cell can be seen to remain on the cantilever as it is retracted and it maintains a rounded shape. Arrows highlight cell position. Scale bar is 20 µm.

**Figure 2:**
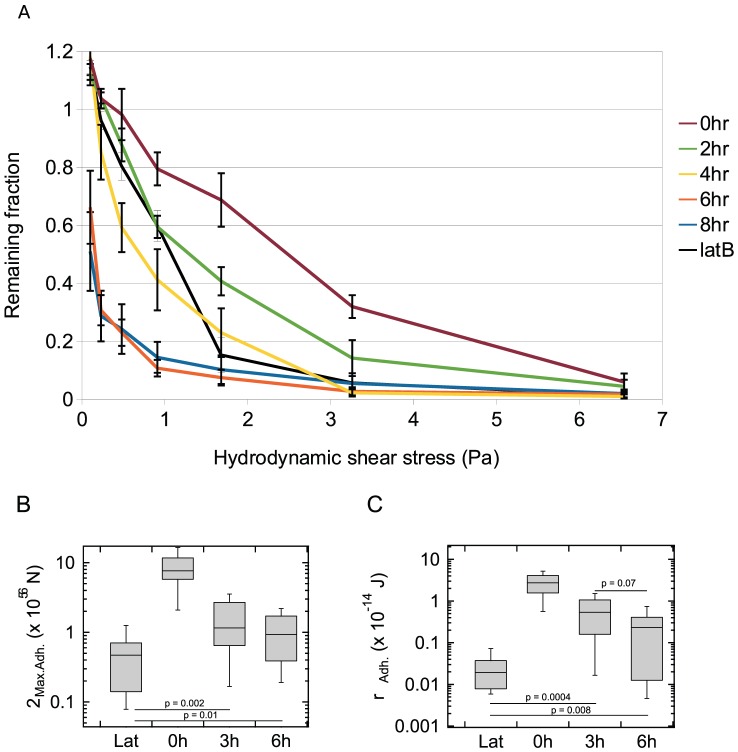
Decrease in adhesion during early development of *Dictyostelium discoideum*. **A.** Microfluidic assay: the remaining fraction of cells after 40 minutes in chambers 2–8 is shown for cells that had developed for varying lengths of time. latB refers to 0 hr cells that were treated with 10 µM latrunculin B for 30 minutes to disrupt the actin cytoskeleton and assayed in the presence of the drug. Average of at least 5 independent experiments. The bars indicate the S.E.M. **B.** Single cell adhesion force assay of cells during early development. Box plot of the distribution of maximum adhesion forces F_Max.Adh_ of individual single cells, where the bottom and the top of the box represents the first and the third quartiles, and the band corresponds to the median. Cells were developed for 0 h (*n* = 33), 3 h (*n* = 47), 6 hr (*n* = 31). Top whiskers are at 90% and bottom whiskers are at 10% of the distribution. Latrunculin B treated 0 hr cells (Lat) (*n* = 27) were assayed in the presence of 10 µM latrunculin B to disrupt the actin cytoskeleton. **C.** Work of adhesion W_Adh._ measured for the same cells. Nonparametric statistical hypothesis test Wilcoxon rank-sum test were used for significance.

Using a microfluidic shear-flow device previously described in [9], we determined the kinetics of detachment of cells during early development. The strength of adhesion to untreated borosilicate glass was estimated by counting the number of adherent cells as a function of time of exposure to a range of hydrodynamic shear stress [7], [8], [20], [21]. While the absolute forces of adhesion are difficult to estimate in this assay due to variability in the shape and orientation of cells in the device, the assay allows one to judge the relative adhesiveness of different cells. Independently, we also used the cantilever of an AFM to hold a cell and measure the forces needed to detach it from a glass surface (Fig. 1) (Single Cell adhesion Force Spectroscopy, SCFS) [22]–[26]. Both assays found that substratum adhesion decreased dramatically during the first 0–8 hr of development (Fig. 2). By 6 hrs the shear stress necessary for detachment of half the cells in 40 minutes decreased about 10 fold (Fig. 2A). Likewise, both the adhesion force F_Max.Adh_ and the work of substrate adhesion W_Adh._ were reduced about 10 fold after 6 hr of development (Fig. 2B and 2C).

Disruption of the actin based cortex by treating 0 hr cells with 10 µM latrunculin reduced cell substratum adhesion 100 fold as measured by single cell force spectroscopy (SCFS). This treatment also decreased substratum adhesion significantly in the microfluidic assay (Fig. 2A), showing the involvement of the cytoskeleton in cell-substratum adhesion.

### Developmental regulation of substratum adhesion

**Figure 3:**
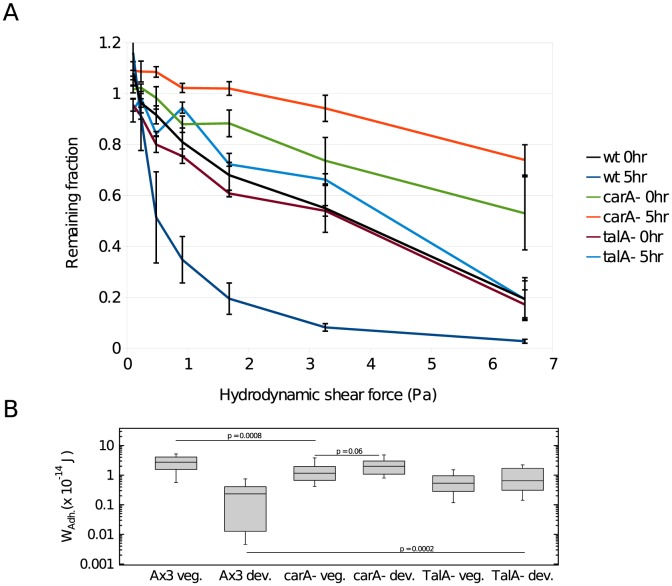
Developmental regulation of the decrease in adhesion. **A** Wild type (AX3) and *carA* ^−^ cells lacking the cAMP receptor after 0 and 5 hr of development were analyzed with the microfluidic assay for the remaining fraction of cells after 40 minutes. **B** Work of adhesion (W_Adh_.) of wild type (AX3), cells lacking the cAMP receptor (*carA* ^−^) or cells lacking talin (*talA^−^*) was measured after 0 hr (veg *n* = 36) and 6 hr (dev *n* = 30 for *carA^−^*, *n* = 34 for *talA^−^*) of development. Nonparametric statistical hypothesis test Wilcoxon rank-sum test was used for significance.

Many of the changes in transcription during early development are dependent on cAMP signaling between the cells [27]–[29]. Signaling is dependent on the cAMP receptor CAR1 that accumulates on the surface of cells during the first few hours of development. Almost no development occurs in cells lacking CAR1 [30]. To determine whether the decrease in substratum adhesion during early development was the consequence of starvation or was developmentally controlled, we analyzed cells carrying null mutations in *carA* (Fig. 3 and Fig S1A: Supple. PLoS.doc). While both the microfluidic and the single cell force assays showed the dramatic decrease in substrate adhesion after 5–6 hr of development in wild type cells, no decrease in adhesion was seen during development of the *carA* ^−^ cells. In fact, the microfluidic assay indicated a slight increase in adhesion when the mutant cells had been starved for 5 hr. It appears that the decrease is a developmentally regulated event.

### Adhesion in cells lacking talin

Substrate adhesion of 0 hr cells of a *talA* ^−^ strain was almost identical to that of wild type cells in the microfluidic assay but significantly reduced as measured by SCFS (Fig. 3 and Fig. S1A: Supple. PLoS.doc). After 5–6 hr of development, cell substrate adhesion increased slightly relative to *talA* ^−^ 0 hr cells as measured by both assays. Thus, both assays reveal that the adhesive properties of *talA* ^−^ cells do not change significantly during the first 5–6 hr of development. Consequently, our results show that cell substratum adhesion is significantly stronger in cells lacking TalA than in wild type cells after 5–6 hr of development.

### Developmental adhesion in cells lacking SibA or SadA

**Figure 4:**
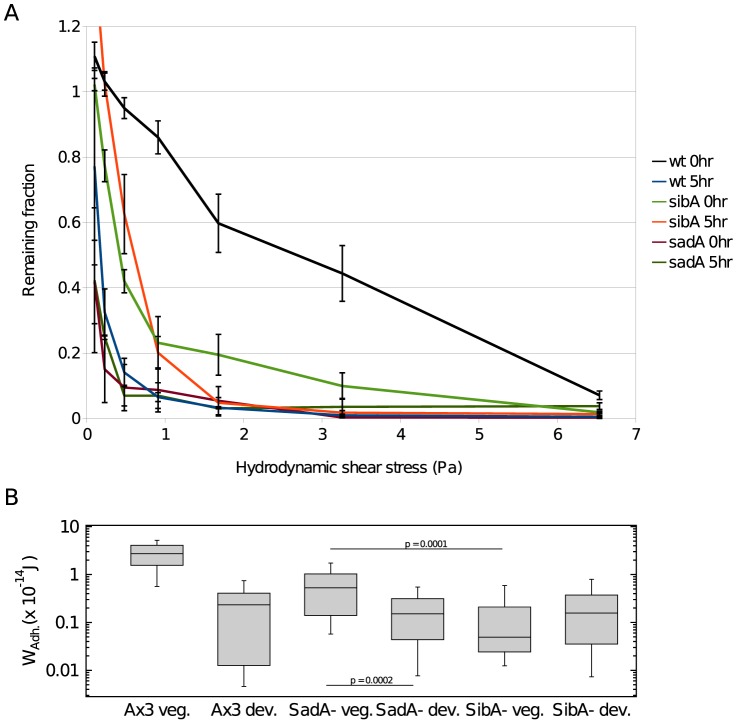
Substratum adhesion in cells lacking *sibA* or s*adA*. **A** Cell-substratum adhesion of wild type Ax3, *sibA* and *sadA* null cells at either 0 or 5 hr of development as measured by the microfluidic assay from the remaining fraction of cells after 40 minutes. **B** Work of adhesion W_Adh._ of wild type (AX3) and cells lacking either *sibA* or *sadA* was measured after 0 hr (*n* = 29 and 41) and 6 hr (*n* = 45 and 41) of development. Significance was judged from the Wilcoxon-rank-sum test.

0 hr cells lacking either SadA or SibA have much reduced substratum adhesion whether measured by the microfluidic assay or the single cell force assay (Fig. 4). The single cell force assay indicated that adhesion dropped further when *sadA* ^−^ cells were developed for 5–6 hr, but adhesion of *sibA* ^−^ cells did not change significantly. By 5–6 hr of development, substrate adhesion in wild type cells had decreased such that it was not significantly different from that in the *sibA* ^−^ or *sadA* ^−^ cells as Wilcoxon-rank-sum test determined p values of ≥0.45 show (Fig. 4B for W_Adh._; Fig. S1B for F_Max.Adh._: Supple. PLoS.doc). It would appear that the strength of substratum adhesion in 5–6 hr developed cells is not dependent on either SibA or SadA.

## Discussion

We have measured two different properties of cell substrate adhesion: resistance of the cells to hydrodynamic shear stress and their resistance to vertical detachment. Both methods differ in the direction of the applied forces – for the microfluidic devices forces on a cell are parallel to the surface the cell adheres to and for the SCFS the forces are verticals to this plane. Under a variety of conditions these two independent assays gave very similar results indicating that they are meaningful characterizations of cell substratum adhesion. Cells were dislodged in the SCFS assay at forces of approximately 5 nN while half the cells were dislodged by shear stress at forces of approximately 1 nN given a cell radius of 10 µm. Of course, the hydrodynamic forces could act over a much more prolonged period than the cantilever retraction. Both the microfluidic shear stress assay and the SCFS assay showed that cell substratum adhesion decreased at least 10 fold during the first hours of development for wild type cells and that this decrease was dependent on the cells being able to respond to pulses of cAMP (Fig. 2 and 3). While the microfluidic assay indicated that cell substratum adhesion was somewhat stronger in cells lacking the cAMP receptor than in wild type cells, the SCFS assay found little difference. This slight discrepancy might indicate that the accuracy of the microfluidic assay is reduced when fewer than half the cells are dislodged in the chamber with the highest shear stress. Likewise, the microfluidic assay loses resolution when characterizing cells with greatly reduced cell substratum adhesion such as seen with the *sibA* ^−^ and *sadA* ^−^ mutants (Fig. 4). In those cases the SCFS assay may be more reliable.

We confirmed that cell substratum adhesion was lower in 0 hr cells lacking either SibA or SadA than in 0 hr wild type cells [11], [18]. These membrane proteins may facilitate close contact of the cell surface with the substratum that favors adhesion. A possible explanation for the decrease in cell-substratum adhesion during early development is down-regulation of SibA or SadA proteins. However, previous work found that the mRNA for SadA increases between T = 0 hr and T = 4 hr and only decreases later [33]. So it is unlikely that there is a significant drop in SadA during early development. The mRNA for SibA decreases as soon as development is initiated but it is not clear that the protein decreases during the first 5 hrs [33]. Testing this hypothesis would require direct quantification of the levels of these proteins which is not possible at this time.

During the first few hours of development, cell-substratum adhesion decreased markedly in wild type cells such that there was no major difference in substratum adhesion of developed wild type and *sibA* ^−^ or *sadA* ^−^ mutant cells. This suggests that the presence or absence of SibA or SadA is irrelevant for substratum adhesion during later steps in development as the cells migrate to aggregation centers, circle around and finally sort out prestalk cells from prespore cells [31], [32].

Finally, we also measured substratum adhesion of cells lacking talin (Fig. 3). We found that *talA* ^−^ null cells were less adhesive than wild type cells at T = 0 hr but not as dramatically as reported previously [13], [19]. The earlier studies were carried out in the growth medium HL-5 which contains sufficient glucose and amino acids to significantly reduce cell-substratum adhesion (Loomis et al., 2012). The present studies were carried out in buffer without either glucose or amino acids where adhesion is stronger. Surprisingly, both the microfluidic and the SCFS adhesion assays revealed that these mutant cells do not change their adhesive properties significantly during the first 5–6 hr of development. This is in sharp contrast to wild-type cells in which adhesion drops 10-fold during the first few hours of development. Unlike mammalian cells, where talin is found in high concentrations in focal adhesions, the role of the talin homolog in *Dictyostelium* cells is unclear and we can only speculate on its precise function. One hypothesis that is consistent with our observations is that talin increases adhesion in 0 hr cells but is inhibitory during development possibly as the result of affecting the rigidity of the membrane since talin has been previously shown to couple force generation to cellular morphogenesis (Tsujioka et al. 2012). Alternatively, the *talA* ^−^ mutant cells might not initiate the portion of the developmental program that reduces adhesion during early development and thereby retain adhesion more than wild type cells.

In mammalian cells, single receptor-ligand interactions - such as those between surface membrane proteins and extracellular matrix components - can generate forces up to 200 pN [34] and cooperativity within focal adhesions can increase the adhesion forces several hundred fold [24], [35], [36]. We found single steps during the detachment of cells with mean values of about 100 pN for wild type 0 hr cells (Fig. 1A). However, it is unlikely that these represent unbinding events from extracellular matrix components since, for each measurement, the cells were repositioned onto pristine surface areas where no cells had attached before. In the microfluidics assay secreted proteins should be continuously flushed away. Using this assay we found little difference in the strength of cell substratum adhesion when 0 hr cells were deposited on untreated glass, silanized glass, serum albumin coated glass or polystyrene [9]. Adhesion appeared to be equally strong on hydrophobic or hydrophilic surfaces. Since the interaction of the cells with the substrate did not appear to be mediated by ionic or hydrophobic bonds and covalent bonds were not formed, we suggested that Van der Waals attraction forces between their surfaces and the substratum might hold the cells. Moreover, the strength of adhesion was reduced by addition of monomeric sugars or amino acids to the buffer, which is consistent with a major role of surface glycoproteins in substratum adhesion. Furthermore, we showed that treatment of vegetative cells with N-acetylglucosaminidase or α-mannosidase also reduced the strength of cell substratum adhesion as measured by either SCFS or microfluidic shear stress (see Figure S2; [9]. Thus, both assays indicated a role for surface glycoproteins in substratum adhesion.

The strength of Van der Waals attractive forces depends on the distance between the objects and increases dramatically as the cell surface approaches the substratum [37]. The membrane on the surface of cells is not smooth but is wrinkled and highly dynamic such that regions closely applied to the substratum change continuously and the total area that can generate Van der Waals forces will depend on the properties of the cytoskeleton [10], [38]. When the cytoskeleton is disrupted by latrunculin inhibition of F-actin, cells have much lower adhesion to the substrate (Fig. 2). Mutations in genes affecting a variety of cytoskeletal components would be expected to affect substratum adhesion. In fact, *sadA* ^−^ cells were found to have aberrant F-actin organization in the cytoskeleton and had a rough surface [18].

In summary, we find strong agreement between two assays addressing surface adhesion of *Dictyostelium* cells. Similar results were found for the adhesive changes during early development, the effects of latrunculin, treatment of the cells with enzymes that can hydrolyze N-linked oligosaccharides on surface proteins, and the consequences of loss of TalA, SibA or SadA. Slight differences can be attributed to the different force regimes the cells are exposed during detachment in the two assays. Cell substratum adhesion is clearly a complex process with different mechanisms working at different size and time scales, however, these assays seem to capture the relative strength of adhesion and further define the critical components.

## Methods

### Cell culture

Wild type (AX3) and mutant strains were grown axenically in HL5 medium [39]. To facilitate computer recognition of individual cells in the flow assay, we transformed these cells with a H2Bv3-RFP construct, in which Red Fluorescent Protein (RFP) with a nuclear localization marker was driven by the actin 15 promoter [9]. Fresh inocula were prepared from lyophilized stocks every few weeks and used in experiments for up to a month. Since we found innate substratum adhesion to be quite sensitive to prior growth conditions, we worked exclusively with cells growing exponentially in suspension in filter sterilized HL5 medium that had not exceeded a density of 2×10^6^ cells/ml. Cells in the exponential phase of growth were deposited on plastic petri dishes in HL5 for 18 hr to allow multinucleated cells to divide to cells of uniform size before measurement of substratum adhesion in the microfluidic device and with SCFS. The mutant strains used in this study have been previously described: *carA* ^−^ [40], [41]; *sadA* ^−^ [18]; *sibA* ^−^ [16] and *talA* ^−^ [19].

Development was induced by washing the cells free of media and resuspending them at 10^7^ cells/ml in 20 mM sodium potassium phosphate buffer pH 6.4 with 200 µM calcium. The cells were shaken and 50 nM cAMP pulses were added every 6 minutes after the first 2 hr.

### Microfluidic assay

Cells were suspended in 20 mM sodium potassium phosphate buffer pH 6.4 with 200 µM calcium at 5×10^5^ cells/ml. A drop of the suspension was placed on a borosilicate glass cover slip and the microfluidic device lowered over the cells in the absence of flow and held by vacuum.1×2 cm microfluidic devices with 8 chambers connected with varying resistance to the outlet were formed in silicon elastomer PDMS by soft lithography [9]. The arrangement of channels and chambers ensured that the flow rate doubled from one chamber to the next generating a 128 fold range in hydrodynamic shear. The input pressure was set at 30 inches water resulting in a flow rate of ∼18 mm/sec in chamber 5. The shear stress varied from 0.05 Pa in chamber #1 to 6.2 Pa in chamber #8. After allowing the cells to settle for 10 minutes, buffer was allowed to flow through the device for 40–60 minutes. The number of cells in a chamber was determined microscopically with a 10× objective every 2 minutes. Cells were counted by a customized program that recognizes RFP in their nuclei. Chamber #1 was considered a settling pond for cells washed down from the upstream resistence channels and settling in the low flow chambers [9]. Results from this chamber were not included in subsequent analyses. In some cases the final cell count in chamber #2 was slightly greater than the initial cell count due to settling of cells from the upstream channels, however, the effect was minimal.

### Single cell adhesion force assay

Tipless cantilevers (Arrow TL2; NanoWorld) with a mean resonance frequency f_o_ = 6 kHz in liquid and a mean spring constant of k = 0.03 N/m were used (thermal noise calibration [42]) in an AFM (Asylum MFP-3D, Asylum Research, Santa Barbara, CA, USA) with increased z-range of 30 µm mounted on an inverted Olympus microscope (IX 51 with 20 or 40× LUCPLFLN objectives). The adhesion of the cantilever to *Dictyostelium* cells was increased by a treatment with polyphenolic adhesive protein mixtures (Cell-Tak, BD Bioscience). In each experiment, the treated cantilever was gently lowered onto a *Dictyostelium* cell. After 60 seconds of contact with 1 nN force, the cell was lifted off until free from the substratum. Then it was attached at a new position on the glass slide with a force of 500 pN and held there for 30 seconds before detaching with a pulling speed of 2.5 µm/s. Contact times, contact forces and pulling speeds were kept constant throughout all experiments and were chosen to allow cells to adhere and not be ruptured (Fig. S3). The attachment-detachment cycle was repeated every 60 seconds for 5 to 10 cycles. A customized MATLAB program was used to calculate the maximum adhesion force F_Max.Adh._ and the work of substrate adhesion W_Adh._ (Fig. 1A). Statistical evaluation was obtained with IGOR Pro Software (Version 6.2.2, Wavemetrics) using the nonparametric Wilcoxon rank-sum hypothesis to obtain p-values. Measurements were taken over a 3 hr period to get a sufficient number of single cell events (*n*≈30).

To visualize cells on the cantilever during the attachment-detachment cycle we used a mirror-based side view with bright-field microscopy on an Olympus IX81 microscope (CellHesion 200, JPK Instruments). Cells could be seen to maintain a rounded shape throughout the attachment-detachment cycle (Fig. 1B; movies S1–3).

## Supporting Information

Figure S1 **F_Max. Adh._ for** ***carA*** **^−^ and** ***talA*** **^−^,** ***sibA*** **^−^ and** ***sadA*** **^−^.**
(EPS)

Figure S2 **F_Max. Adh._ for glycosidase treatment.**
(EPS)

Figure S3 **SCFS-Parametrisation.**
(EPS)

Movies S1 **Top-view bright field, epifluorescence and side-view bright field of AFM-SCFS.** Text to supplemental Movies.
(MOV)

Movie S2 **Top-view bright field, epifluorescence and side-view bright field of AFM-SCFS.** Text to supplemental Movies.
(MOV)

Movie S3 **Top-view bright field, epifluorescence and side-view bright field of AFM-SCFS.** Text to supplemental Movies.
(MOV)
